# Bilateral anterior dislocation of the shoulders at the start of a backstroke competition

**DOI:** 10.1007/s10195-011-0176-5

**Published:** 2012-02-09

**Authors:** Fayçal Dlimi, Abdelkarim Rhanim, Abdou Lahlou, Mohammed Kharmaz, Mohammed Ouadghiri, Ahmed El Bardouni, Mohamed Saleh Berrada, Mustapha Mahfoud, Moradh El Yaacoubi

**Affiliations:** Department of Orthopaedic Surgery and Traumatology, University Hospital Center, Ibn Sina, Rabat, Morocco

**Keywords:** Anterior dislocation, Bilateral dislocation, Closed reduction, Shoulder dislocation, Swimming

## Abstract

Bilateral anterior dislocation of the shoulders is very rare. A 20-year-old man presented with bilateral anterior shoulder dislocation as a result of a diving incident. He complained of pain and restriction of movement in both shoulders with abducted and externally rotated arms. Radiographs revealed that the shoulders were dislocated. The patient was treated with closed reduction and was able to resume swimming 3 months later. To our knowledge, this is the first bilateral anterior dislocation of the shoulders during a backstroke swimming competition that was caused by this mechanism of injury. The rarity of this lesion and its uncommon mechanism prompted us to relate this observation.

## Introduction

Although anterior shoulder dislocation is the most common major joint dislocation encountered by emergency physicians, bilateral glenohumeral dislocations are rare and almost always posterior [1]. Such dislocations are usually caused by sports injuries, seizures, electrical shock, or electroconvulsive therapy [2, 3]. However, simultaneous bilateral anterior shoulder dislocation (BADS) is very rare: only about 30 cases have been described in the literature [2, 4–6]. We report a rare case involving traumatic bilateral dislocation of the shoulders as a result of a diving incident.

## Case report

**Fig. 1:**
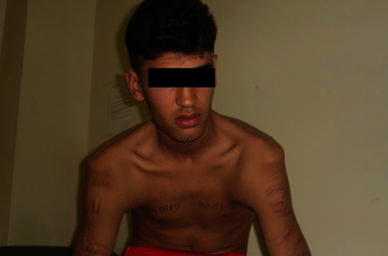
Clinical appearance of bilateral anterior dislocation with squaring of the shoulders

**Fig. 2:**
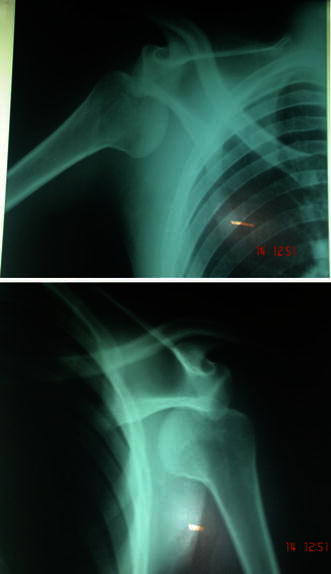
Radiographs of the shoulders demonstrate bilateral anterior dislocation

**Fig. 3:**
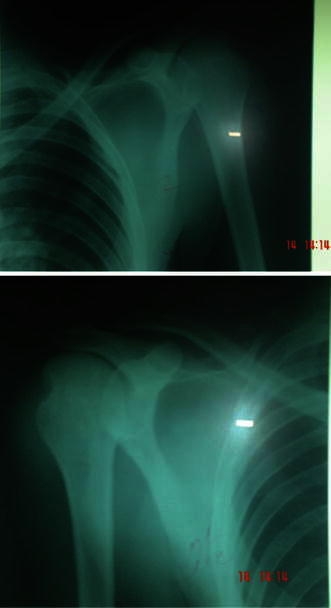
Check X-rays showing reduced joints

**Fig. 4:**
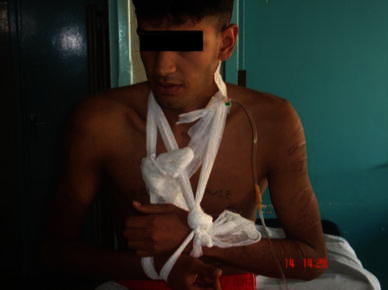
Clinical appearance after reduction

A 20-year-old man experienced bilateral anterior dislocation of the shoulders while diving at the start of a backstroke swimming competition. The backstroke start is the only start that takes place in the water. For the takeoff, the swimmer pushed his hands away from the block, swung his arms around sideways to the front, and threw his head to the back. After the start, he was completely underwater, but soon reappeared on the surface and could not swim. He suddenly felt that his shoulders were going out of place and was unable to continue the race. He was immediately rushed to the emergency department complaining of acute bilateral shoulder pain and stiffness. His arms were abducted and externally rotated. The patient had no history of seizure, epilepsy, alcohol intake, or previous shoulder dislocation. None of his family had any history of hyperlaxity disorders, epilepsy, or convulsions. Physical examination revealed fullness over the anterior aspect with a bilateral flattened contour of the shoulders below the tip of the acromion; however, luckily, the patient did not suffer from any neurological or vascular injuries (Fig. 1). Radiographs of his shoulders showed bilateral anterior dislocation (Fig. 2). These were reduced by closed manipulation, confirmed by radiographs, and the arms were immobilized in adduction and internal rotation for 3 weeks (Figs. 3, 4). The patient received regular physiotherapy and was able to resume competitions 3 months after. At one-year follow-up, the outcome was satisfactory and the patient had an excellent range of motion in both shoulders.

The patient consented to the publication of this case report.

## Discussion

Anterior shoulder dislocation is the most common major joint dislocation encountered in the emergency department. Its injury mechanism is forced extension, abduction, and external rotation. Anterior dislocation of the shoulder may occur in a violent contraction of the shoulder muscles or a direct blow to the posterior aspect of the shoulder. Because of the position naturally adopted by the upper extremity during a fall, unilateral anterior dislocation of the shoulder is common. Bilateral glenohumeral dislocations are rare and almost always posterior [1, 2, 4]. However, BADS is very rare: a review of the literature revealed about 30 reports of bilateral anterior shoulder dislocations, 15 of which were of fracture-dislocation. Most were due to violent trauma or electrocution; the remaining few were attributed to epileptic or hypoglycemic seizures [7, 8]. BADS was first described in 1902 in a patient with muscular contraction caused by a camphor overdose [2, 9]. Sports injuries, seizures, electrical shock, electroconvulsive therapy, drug overdose, neuromuscular disorders, and psychiatric disturbances have been implicated [2, 3, 8, 10–12]. Only one case was connected with a sporting activity: a water skier lost control at high speed and was thrown violently across the surface of the water [13]. However, it was not reported whether the shoulders were dislocated by the forward pull of the tow-rope or during the fall, because it is not easy to imagine how a fall on the surface of the water would dislocate both shoulders. Our patient was a promising young swimmer who dislocated his shoulders while diving at the start of a backstroke race. This is the only competition swimming style that starts in the water. In backstroke, the arms contribute most of the forward movement. The stroke consists of two main parts: the power phase and the recovery. The dislocation usually occurs when the swimmer has the arm in the cocked position associated with hyperextension of the shoulders. The force can be strong enough to rupture the anterior capsule and glenohumeral ligament complex, resulting in anteroinferior dislocation. In addition to that, our young, inexperienced swimmer was unfortunately unfocused and highly stressed before the start of the race. Clinical diagnoses of dislocation types and associated injuries may be inaccurate without imaging [2, 10, 11]. Delayed diagnosis is not uncommon in bilateral shoulder dislocations resulted from electric shock or trauma [11, 14, 15]. Surgery is reserved for recurrent cases, which are mostly seen in patients who are <40 years of age [4, 16].
